# Supplementary material for: Surrounded by Kindred: Spermophilus major Hybridization with Other Spermophilus Species in Space and Time
Source: Biology (Basel). 2023 Jun 17;12(6):880. doi: 10.3390/biology12060880 (PMC10295132; doi:10.3390/biology12060880)
Supplement: Supplementary file 1 [file biology-12-00880-s001.zip › Tukhbatullin&Brandler_Tables_S2 and S3_Supplementary.pdf]

Surrounded by kindred: *Spermophilus major* hybridization with other *Spermophilus* species in space and time

Andrey Tukhbatullin, Oleg Ermakov, Svetlana Kapustina, Vladimir Starikov, Valentina Tambovtseva, Sergey Titov, and Oleg Brandler

Supplementary Materials

Table S2: The primers and amplification reaction conditions.

Markers	Praimers		Denaturation		Annealing		Extension		Number of cycles	References
	Name	Sequence	t° C	time	t° C	time	t° C	time		
mtDNA										
CR PCR	MDL1	TCCACCTTCAACTCCCAAAGC+M13F*	95	30 s	58	45 s	72	1 min	35	[49]
	H00651	TAACTGCAGAAGGCTAGGACCAAACCT+M13R*								[98]
Cytb PCR	L14725	TGAAAAAYCATCGTTGT+M13F*	95	30 s	48	40	72	1 min	35	[99]
	H15915	TCTTCATTTYWGGTTTACAAGAC+M13R*								[100]
Sequencing reaction	M13F	TGTAAAACGACGGCCAGT								[101]
	M13R	CACAGGAAACAGCTATGACC								
nDNA										
8iSmcY	Smc8D	ATGCTCTCGTGGGGATGAAG	95	20 s	56.3	40	72	20 s	35	[53]
	Smc9R	ACAGGCATGTTGAAGTAGTC								
BGN	BGN-f	CTCCAAGAACCACCTGGTG	95	40 s	61	50 s	72	50 s	35	[66]
	BGN-r	TTCAAAGCCACTGTTCTCCAG								
PRKCI	PRKCI-f	GGGTAATAGGAAGAGGAAGTT	95	40 s	54	50 s	72	50 s	35	[67]
	PRKCI-r	CCAACAAGGAAAGGATGAT								
c-mic	S91	RRAGCCTCATTAAGTCTTAGGTAAGAA	95	45 s	60	45 s	72	1.5 min	37	[69]
	S92	CCMAAGACYCAGCCAAGGTTGTGAGGT								
rejected nDNA markers										
GBA	GBA-f	AAAAGCTTCGGCTACAGCTC	95	40 s	56	1 min	72	50 s	35	[66]
	GBA-r	TCCCTTCACTTTCTGGAACTTC								
BCR	SMD1	CACATTCCACTGACCATCAAC	95	30 s	60	50 s	72	1 min	30	[25]
	D3	AGTGGCTGAGTGGACGATGA								
MGF	MGF-f	ATCCATTGATGCCTTCAAGG	95	40 s	56	1 min	72	1 min	35	[66]
	MGF-r	CTGTCATTCCTAAGGGAGCTG								
THY	THY-f	CATCAACACCACCATCTGTGC	95	40 s	49	1 min	72	1 min	35	[68]
	THY-r	CACTTGCCACACTTACAGCT								

* Specific primers were modified in 5' ends with complementing by sequence primers (M13f and M13r) for polymerase chain reaction (PCR).

Table S3: The nucleotide sequences of the *i6p53* ID repeats

Species	Sequence
S. major	TTTTTGTTTTTTTTTTT
S. b. heptneri	TTTTCGTTTTTTTTTTT
S. b. iliensis	T
S. fulvus	TTTTTGTTTTTTGTTGTTGTTGTTGTTGTTGTTGTTTTTTT
S. erythrogenys	TTTTTGTTTTTTTGTTTGTTTGTTTGTTTTTGTTTTTGTTTT
*S. pygmaeus**	TTTTTGTTTTTTTGGGGTTTTTTTTTGTTTGTTTGTTTGTTTGTTTGTTTTGTTTTGTTTTGTTTTGTTTTGTTTT
	TTTTTGTTTTTTTGGGGTTTTTTTTTGTTTGTTTGTTTGTTTTTTTGGTTTTGTTTTGGTTTTTGTTTTTTTTTTT
	TTTTTGTTTTTTGGGGGGTTTTTTTTGTTTGTTTGTTTGTTTTTTTGGTTTTGTTTTGTTTTTTGTTTTTTTTTT
	TTTTTGTTTTTTTGGGGTTTTTTTTTTTGTTTGTTTGTTTGTTTGTTTTTTTTTGTTTTGTTTTTTTGTTTTTTTTTTT

* - Four types of the *i6p53* ID repeats have been found in this species.
